# Supplementary material for: Community-based adaptation of early adolescent skills for emotions for urban adolescents and caregivers in New York City
Source: Glob Ment Health (Camb). 2025 Sep 10;12:e107. doi: 10.1017/gmh.2025.10045 (PMC12509165; doi:10.1017/gmh.2025.10045)
Supplement: Wong et al. supplementary material [file S2054425125100459sup001.docx]

# Supplemental File 1 - RECAPT criteria

# A) Set-up

| Criterion 1: Definition of the target population | |
| --- | --- |
| **Category** | **Results** |
| Target group | Urban (largely ethnic minorities, first/second generation immigrant, and low-income families) English-speaking caregivers and adolescents (ages 10-17) in New York City (NYC) |

| Criterion 2: Team and roles | | | | | |
| --- | --- | --- | --- | --- | --- |
| **Team member** | **Gender** | **Disciplinary background** | **Level, experience, other relevant information** | **Cultural characteristics** | **Role** |
| AB | M | Clinical psychologist | Professor  Experience leading research in cultural adaptation internationally and in NYC; specialized in trauma, migrant mental health, and task-sharing mental health interventions. | American | Supervisor |
| JW | F | Psychology | Research associate  Experience working with immigrant groups and adapting task-sharing mental health interventions. | Chinese (Hong Kong) | Led research project, discussant, data collection and analysis for all stages; Trained as EASE trainer in the current study |
| TX | F | Psychology | MA candidate  Experience working with ethnically minoritized and immigrant adolescents in community settings and task-sharing mental health interventions. | Chinese American | Led research project, discussant, data collection and analysis for all stages; Trained as EASE trainer in the current study |
| CS | F | Design | Designer  Experience with design strategy, graphic design, and developing adaptations for Global Mental Health task-sharing mental health interventions. | Indian | Discussant, adaptation illustrator |
| JC | F | Psychology | PhD candidate  Experience with adaptation work related to task-sharing mental health interventions, and providing mental health support to vulnerable populations. | Indian | Discussant, data collection and analysis for all stages |
| NI | F | Design | Designer  Experience in design strategy, visual communication, branding, and working with Hispanic communities on mental health initiatives. | Mexican | Discussant, adaptation designer, data collection, research, and analysis for all stages |
| DS | F | Psychology | MA candidate  Experience working as a Research Assistant in a global mental health lab and a lab for human attachment. | Chilean | Zoom technological facilitator |

# B) Formative research

| Criterion 5: Formative research methods | |
| --- | --- |
| **Category** | **Results** |
| Literature review | Desk review on different relevant topics (e.g., facilitators and barriers to the use of adolescent mental health services in the US, scalable task-sharing mental health interventions, cultural adaptation of task-sharing interventions, etc.) |
| Qualitative methods | We sought to learn about the mental health challenges urban adolescents in NYC face, whether Early Adolescent Skills for Emotions (EASE) would be viable for reducing the distress they are experiencing, and collect feedback on the ways in which EASE may be modified to better suit the needs of this population.  In the qualitative part of the study, discussants led seven focus group discussions (FGDs) with adolescents (n=18) and three FGDs with caregivers (n=12). The adolescent FGDs were divided into two subgroups of younger and older adolescents at each session so that two similarly-sized adolescent FGDs were run in parallel, with two members from the research team in each subgroup as facilitators. Although EASE is intended for 10 to 15-year-olds, we included older adolescents (16 and 17-year-olds) in our FGDs to better understand the range of mental health challenges that may emerge over the continuum of adolescence, as older adolescents can provide insights into the psycho-emotional needs of younger adolescents, and younger adolescents can better prepare for challenges emerging later in adolescence.  The average duration of the focus group discussions was 1 hour and 30 minutes. For each group of younger and older adolescents, one research staff member led the group discussion while the other one took detailed notes. The notes were coded by researchers JW, TX, and JC to identify themes related to adolescents’ and caregivers’ perceptions of mental health and items pertaining to the accessibility and relevance of EASE. Within the FGDs, the following categories were identified as relevant to urban adolescents’ experiences of mental health in NYC: (a) Lack of understanding of distressing emotions, (b) lack of knowledge of how to manage and respond to distressing emotions, (c) feeling the need to hide their emotions, (d) connection with physical health and somatic experiences, (e) considering distress as related to lack of purpose, meaning and connection. The following categories were identified as essential to intervention accessibility and relevance: (a) Promoting inclusivity and sharing in vulnerable spaces, (b) desire for knowledge-based explanations of psychological benefits, (c) building purpose, meaning, connection and identity.  Additional contextual stressors were identified by adolescents and caregivers: (a) Gender norms in caregiving, (b) role of digital technology, (c) peer relationships, (d) economic pressures on the family.  Specific questions pertaining to these categories were asked during the cognitive interviews at the final FGD to collect additional feedback. Meetings with city stakeholders and community organizations framed treatment goals in terms of (a) socioemotional learning and (b) community building. |
| Quantitative methods | In the quantitative part of the study, we collected adolescents’ (n=18) ratings of all the EASE activities based on how (1) helpful and (2) engaging the adolescents found them out of 5 after completion of the entire program. The average scores for the individual ratings and the average scores across both ratings are reported in **Table 2** of the manuscript. |

| Criterion 6: Target symptoms, syndromes, needs, and context | | | |
| --- | --- | --- | --- |
| **Category** | | **Results** | **Source** |
| Cultural concepts of distress | Idioms of distress, specific target symptoms | Emotion-related symptoms: sadness and depressed mood, irritability, anxiety, emptiness, lack of will to live, boredom, shame  Cognitive symptoms: impaired performance (academic, social) and concentration, confusion, worrying about the future, comparison with others  Behavioral symptoms: change in facial expressions and body condition (e.g. frowning, tense muscles, appetite), crying, shouting, withdrawal (isolation), avoidance (sleeping too much)  Coping: Sleeping, listening to music, playing video games, exercise and sports | FGDs, cognitive interviews |
|  | Explanations of distress | Lack of understanding of distressing emotions   - Adolescents cited that they never learned about how to explicitly identify their emotions with nuanced vocabulary, whether in their families, schools, or broader culture.   Lack of knowledge of how to manage or respond to emotions   - Adolescents cited that they sometimes felt controlled by their emotions and had automatic reactions to events that made them sad or angry.   Feeling the need to hide their emotions   - Adolescents cited that they often held a neutral “poker face” that was incongruous to how they felt inside, especially adolescent males, and that they sometimes did not want others to know their true emotions, and sometimes did not know how to share their emotions productively.   Connection with physical health and somatic experiences   - Adolescents noted that they sometimes saw distress as a function of poor physical health. For example, they would explain feeling down or a lack of motivation due to a lack of sleep in the previous night, or experiencing anger as a physical sensation of heating up in the body.   Considering distress as related to lack of purpose, meaning, and connection   - Both younger and older adolescents cited lack of purpose, meaning, and connection as a reason for feelings of distress |  |
|  | Beliefs about the course of the disorder and help-seeking behavior | Adolescents believed that community-based organizations are important to their socioemotional development, providing a place to be vulnerable with peers and trusted adults. While social situations at school and families could be a source of stress, CBOs are perceived to be a valuable resource for adolescents as a site to learn about themselves and find a sense of connection and belonging within their community. |  |
| Community needs, stigma, and context | Attitudes towards mental health | Promoting inclusivity and sharing in safe spaces   - Caregivers and adolescents noted that hesitancy to express mental health struggles comes from the fear of judgment from peers, even though they did not necessarily see mental health struggles as shameful. They advocated for safe, inclusive environments to share, learn from each other, and grow.   Desire for deeper knowledge-based explanations of psychological benefits   - Adolescents were strongly interested in the mind-body connection and considered mental health as interconnected with physical health. The Adolescents were interested in learning about the physiological basis or scientific rationale behind the strategies, such as the effects of slow breathing on the nervous system.   Building purpose, meaning, connection, and identity   - Both younger and older adolescents wanted not only to decrease distress, but increase feelings of purpose, meaning, and connection in their lives as protective factors against depression. - Adolescents were eager to deepen their understanding of themselves, their own strengths and weaknesses, and to connect their personal identities with society and the outside world. |  |
|  | Specific needs and other relevant contextual information | Access to mental health resources   - Existing school health classes impart knowledge on topics about emotional management. - However, the adolescents said that these topics are only handled in a cursory manner: They seem one-sided from the provider and these spaces only highlight the importance of being aware of one’s emotions, not so much the practical ways of controlling emotions or even creating a safe, shared space for adolescents to come together and build community. - Based on the original WHO EASE manual, the current adaptation covers a wide range of topics related to social emotional learning: Enhancing emotional awareness by expanding emotional vocabulary and encouraging adolescents to express emotions, teaching adolescents strategies to cope with and practically manage big feelings (e.g. slow breathing) and deal with their problems and challenges (e.g. gradually engaging in activities to improve mood and brainstorming solutions to a problem). It also includes collective practice of skills and workbooks for adolescents to continue at home between sessions.   Gender norms   - Caregivers advocated for a feminist perspective in understanding caregiver dynamics and responsibilities. They expressed the lack of explicit attention on the burden and challenges faced by female caregivers. - Caregivers also alluded to how a program like EASE that promotes the development of socio-emotional skills might be particularly beneficial for adolescent males. This suggests a possible preconception or stereotype that adolescent females are more “expressive” and “in tune” with their emotions than are adolescent males.   Digital technology   - Caregivers expressed how digital technology can adversely affect adolescents’ mental health. For example, adolescents can get addicted to social media, spend a lot of time on their gadgets, and isolate themselves from connecting with people around them. Social media may also negatively affect adolescents’ perceptions of themselves as they may judge themselves through comparisons with peers/ influencers they see online. - Adolescents concurred with the problems associated with digital technology on mental health. However, adolescents highlighted how digital technology can positively influence mental health too by providing a space for like-minded individuals to come together and bond virtually.   Economic pressures on the family   - Adolescents cited the economic pressures of the expensive and rapidly rising cost of living in NYC as sources of stress to the family   Peer relationships   - Adolescents expressed that “friendship drama” problems are understood as differences, disagreements, or misunderstandings. Bullying in the NYC context occurs in relation to social media use, exclusion, and peer pressure.   Group dynamics   - Adolescents emphasized that it was important to get to know each other first, build trust, and set boundaries with group rules within a group to make it easier for them to all open up and communicate their feelings. - They also highlighted the importance of bonding with the facilitators: Genuine, honest sharing from facilitators can encourage adolescents to open up. - In the beginning, adolescents tended to share more about happy and silly experiences rather than negative emotions with the group. However, when approached individually by a member of the research team, they were more willing to share. - As the sessions progressed - adolescents got to know each other better and were encouraged by research team members to share - group dynamics shifted such that adolescents were more willing to share negative feelings with the rest of the group. |  |
| Treatment components | Framing treatment goals | Developing social and emotional learning (SEL)   - Promote the development of SEL in adolescents ages 10-15 by aligning EASE with competencies identified by the Collaborative for Academic, Social and Emotional Learning ([CASEL)](https://casel.org/fundamentals-of-sel/what-is-the-casel-framework/)   - The five competencies are: Improving self-awareness, self-management, social awareness, relationship skills, and responsible decision-making   - The CASEL framework is uplifted and integrated into programming by the Department of Youth & Community Development (DYCD) in NYC. It provides guidelines on the skills and attitudes for young people to develop their identities, manage emotions, achieve personal and social goals, empathize with others, establish and maintain supportive relationships, and make sound decisions.   Community-building   - Caregiver and adolescent participants expressed great enthusiasm for the intervention’s community-building approach. They endorsed the promotion of EASE aspects for relationship-building and emotional management through shared lived experiences with participating adolescent peers, CBO staff facilitators, and caregivers. |  |
| Treatment delivery | Delivery format | Barriers to and advantages of use   - Adolescents do not prefer virtual sessions and value face to face interventions to build trust and foster real-life connections. - Community-based setting for adolescents. - Virtual sessions for caregivers to accommodate busy schedules.   Promotion of use   - Can potentially be incorporated in existing adolescent groups within community organizations or afterschool programs. |  |
|  | Surface adaptations | Content   - Need of readily available, age-appropriate icebreakers and refreshing activities throughout the sessions to direct adolescents’ attention to the group. - Caregivers and adolescents expressed the importance of improving the outward appeal of EASE to make the program more engaging for their community. This included incorporating trendy audiovisual materials and more NYC-relevant representations of illustrations/day-to-day life references with which participants can identify.   Language   - Adolescents expressed the need to use more NYC-lingo in EASE sessions to make the program more relevant and engaging for their community and age-group. |  |

#

# C) Intervention adaptation

| Criterion 7: Specific treatment elementsCriterion 8: Unspecific elements and therapeutic techniques | | | | | | |
| --- | --- | --- | --- | --- | --- | --- |
| **Decision-Nr.** | **Mechanisms of action including treatment elements, techniques, delivery, surface** | **Original intervention** | **Cultural Processes related to mechanism of action** | **Cultural/ contextual adaptation** | **Evidence base** | **Quality of evidence** |
| **Adolescent Session 1: Understanding My Feelings (Orientation, externalizing and identifying personal feelings)** | | | | | | |
| 1 | Cultural concepts of distress: Explanations of distress: Lack of understanding of distressing emotions  And  Treatment components: Framing: Treatment goal | Feelings Chart poster displays illustrations expressing ten different facial expressions without labels | Cultural model of rich emotional vocabulary as necessary for better recognition, labeling and communication of nuanced feelings | Addition of a feelings wheel that further breaks down ten original facial expressions in EASE to over 50 emotions popular within local culture (such as “resentful,” “lonely,” “dread”) | FGDs | Strong |
| 2 | Cultural concepts of distress: Explanations of distress: Feeling the need to hide their emotions | Feelings Pot poster and workbook exercise encourages adolescents to draw their emotions in a “pot” | Cultural model of emotions as something to be expressed rather than “bottled up” | Feelings Pot changed to Feelings Canvas to denote free expression of emotions on poster and in workbook activity | FGDs | Moderate |
| **Adolescent Session 2: Calming My Body (Connecting feelings to the body, learning about coping strategies and slow breathing)** | | | | | | |
| 3 | Community needs, stigma, and context: Attitudes towards mental health: Promoting inclusivity and sharing in safe spaces | Body Map poster and activity encourages adolescents to draw physical sensations associated with different feelings on one male outline | Cultural model of importance of reducing shame around different body types | Expanded body map poster outlines four versions of bodies that reflect different genders (including gender neutrality) and body shapes | FGDs | Moderate |
| 4 | Community needs, stigma, and context: Attitudes towards mental health: Desire for deeper knowledge-based explanations of psychological benefits | Slow breathing activity involves facilitators guiding adolescents to breathe slowly from their belly and counting each breath | Cultural model of mind-body connection as important tools for mental wellbeing and using mindfulness to address somatic symptoms | Slow breathing connected to somatic experience through added variations such as focusing on a body part with distress; added a slow breathing infographic to explain how the skill is related to physiological changes | FGDs | Moderate |
| **Adolescent Session 3: Changing My Actions I (Learn how problems and feelings can change actions or behaviors and how to gradually engage in enjoyable activities to improve mood)** | | | | | | |
| 5 | Community needs, stigma, and context: Attitudes towards mental health: Building purpose, meaning, connection, and identity | Storybook discussion of main character’s hobbies | Cultural model of building meaning, purpose, connection and identity | Purpose Diagram activity added for adolescents to reflect on the intersection of what they love, where they excel, where they can make a difference, and how they see the world (Part 1: What I love) | FGDs | Moderate |
| **Adolescent Session 4: Changing My Actions II (Continue learning how to gradually engage in activities to improve mood with a focus on important tasks)** | | | | | | |
| 6 | Community needs, stigma, and context: Attitudes towards mental health: Building purpose, meaning, connection, and identity | Vicious cycle poster and activity involves discussion about how big feelings can stop engagement in enjoyable and meaningful activities and lead one to feel worse | Cultural model of approaching problems with a positive mindset | Concept and activity for “virtuous cycle” added after introduction of “vicious cycle” | FGDs | Moderate |
| 7 | Community needs, stigma, and context: Attitudes towards mental health: Building purpose, meaning, connection, and identity | Storybook discussion of main character doing something he is good at | Cultural model of building meaning, purpose, connection and identity. Bridging between what the adolescent enjoys doing with what they are good at to discover their passion. | Purpose Diagram activity added for adolescents to reflect on the intersection of what they love, where they excel, where they can make a difference, and how they see the world (Part 2: Where I excel) | FGDs | Moderate |
| **Adolescent Session 5: Managing My Problems I (Understanding common problems, learn how to brainstorm solutions to solve common problems and apply the strategy to a personal problem)** | | | | | | |
| 8 | Community needs, stigma, and context: Attitudes towards mental health: Building purpose, meaning, connection, and identity | Managing my problems activity encourages adolescents to pause, brainstorm solutions, and select the best solution to proceed with | Cultural model of approaching problems with a positive mindset | Introduce concept of fixed versus growth mindset and the growth zone. | FGDs | Moderate |
| **Adolescent Session 6: Managing My Problems II (Continue learning how to brainstorm solutions to personal problems, empower participants to learn from each other)** | | | | | | |
| 9 | Cultural concepts of distress: Explanations of distress: Lack of understanding of distressing emotions; Lack of knowledge of how to manage and respond to emotions. | Practicing brainstorming problems and solutions to problems | Cultural model of the need to identify what things in life can or cannot be controlled in order to focus on aspects of emotional distress or problems that can be influenced and managed more effectively. | Circle of control activity added to encourage adolescents to brainstorm what things in life they can influence versus those that are beyond control to better frame the subsequent “Managing My Problems” activity. | FGDs | Moderate |
| 10 | Community needs, stigma, and context: Attitudes towards mental health: Building purpose, meaning, connection, and identity | Practicing brainstorming problems and solutions to problems | Cultural model of building meaning, purpose, connection and identity. Bridging between passions, values, gifts, and possibilities to encourage thinking about one’s place in the world. | Purpose Diagram activity added for adolescents to reflect on the intersection of what they love, where they excel, where they can make a difference, and how they see the world (Part 3 and 4: Where I can make a difference, How I see the world) | FGDs | Moderate |
| **Adolescent Session 7: Brighter Futures (Educate participants on what to expect in the future, improve participants’ confidence in responding to future problems and big feelings)** | | | | | | |
| 11 | Community needs, stigma, and context: Attitudes towards mental health: Building purpose, meaning, connection, and identity | Educate participants on what to expect in the future and improve participants’ confidence | Cultural model of building meaning, purpose, connection and identity. Tying together individual strengths and learned socioemotional skills with community awareness and long-term planning. | Final discussion, sharing and display of adolescents' Purpose Diagrams as a reflection of how adolescents see the ways their passion, values, gifts, and possibilities can contribute to their community and the world around them | FGDs | Moderate |
| 12 | Treatment components: Framing treatment goals: Community-building | Improve participants’ confidence for the future | Cultural model on the importance of building community connectedness in celebrating completion and passing on lessons to future EASE participants | Graduation ceremony with printed diplomas for adolescents, EASE “yearbook” activity to write or draw one page of encouragement to be passed to future EASE adolescents for ongoing book for local community building | FGDs | Moderate |
| **Caregiver Session 2: The Power of Praise (Praising children’s strengths, alternatives to harsh punishment)** | | | | | | |
| 13 | Community needs, stigma, and context: Attitudes towards mental health: Promoting inclusivity and sharing in safe spaces | Caregiver story discusses the main character Kian’s parents worrying about Kian experiencing big feelings and berating Kian with physical punishment and mentions of shame | Cultural model of mental health not being considered as shameful, rather, it is conceived of something inherent to oneself and something to improve on gradually. | Removed associations of shame and physical punishment for mental health. | FGDs | Moderate |
| **Caregiver Session 3: Caregiver Self-care and Brighter Futures (Caregiver strengths and challenges, self-care, and improving caregivers’ confidence in managing difficulties with their children in the future)** | | | | | | |
| 14 | Community needs, stigma, and context: Specific needs and other relevant contextual information: Gender norms | Second and third parts of the caregiver story discuss the ways in which Kian’s parents are trying to support Kian. Particularly, Kian’s mother Fatima is described as being too occupied with making sure her children went to school, had meals, and had clean clothes to wear, while Kian’s father Aban is described to act as a good role model for Kian. The narrative focuses on Fatima worrying about the family dynamic, while Aban is described to be increasingly irritable with things at home. | Cultural model of the need to discuss feelings in a more equitable manner for both caregivers outside of traditional gender stereotypes of dealing with emotions | Encourage discussion about caregiver responsibilities that may or may not be influenced by gender roles by incorporating potential challenges of female caregivers in storyline as well as discussion questions applicable to single parent and non-heterosexual caregivers.  Incorporated a greater male caregiver presence in the storyline and more equal discussion about the feelings and worries of both caregivers.  Encourage discussion about how asking for help may be seen as a way of self-care. | FGDs | Moderate |

| Criterion 9: Surface adaptations | | | | | |
| --- | --- | --- | --- | --- | --- |
| **Decision-Nr.** | **Treatment elements, techniques, delivery, surface** | **Original intervention** | **Cultural / contextual adaptation** | **Evidence base** | **Quality of evidence** |
| **Adolescent Storybook, Posters, Workbook, and Activities** | | | | | |
| 15 | Treatment delivery: Surface adaptations: Content | Birdwatching as main character Kian’s favorite hobby | Skateboarding as main character Kian’s favorite hobby | FGDs | Moderate |
| 16 | Treatment delivery: Surface adaptations: Content | Colorful, talking birds that represent how color can be used to express emotion | Artist in community organization teaches Kian to use different colors on a canvas to express emotion | FGDs | Moderate |
| 17 | Treatment delivery: Surface adaptations: Content  or  Community needs, stigma, and context: Specific needs and other relevant contextual information: Digital technology; Economic pressures on the family; Peer relationships | Students stealing snacks from one another as a form of bullying and poor peer relationships | Cyberbullying, exclusion, financial concerns, and peer pressure included in the storybook as challenges adolescents face in their communities | FGDs | Moderate |
| 18 | Surface: Treatment delivery: Surface adaptations: Content  or  Community needs, stigma, and context: Specific needs and other relevant contextual information: Digital technology | No mention of technology’s impact on adolescents’ lives | Social media is woven throughout the storybook as a way for Kian to arrange activities with friends, a source for him to learn new skills and knowledge (skateboarding tricks), as well as a source of distress with social comparisons and cyberbullying | FGDs | Moderate |
| 19 | Treatment delivery: Surface adaptations: Language | Language considered to be too formal for the adolescents | Colloquial language and local idioms incorporated into the final text | FGDs | Moderate |
| 20 | Treatment delivery: Surface adaptations: Content | Characters in simple clothing, no shoes, plain backgrounds with no color | Characters and settings redrawn to be more representative of NYC, addition of color throughout all materials | FGDs | Moderate |
| 21 | Treatment delivery: Surface adaptations: Content | Adolescents to decorate Feelings Pot with colored pencils | Option of using emoji stickers in addition to colored pencils to decorate Feelings Canvas | FGDs | Moderate |
| **Others** | | | | | |
| 22 | Treatment delivery: Delivery format: Barriers to and advantages of use | 7 in-person sessions for adolescents and 3 in-person sessions for caregivers | 7 in-person sessions for adolescents and 3 virtual sessions for caregivers | FGDs | Strong |
| 23 | Treatment delivery: Surface adaptations: Content  or  Community needs, stigma, and context: Specific needs and other relevant contextual information: Group dynamics | Roleplays to simulate big feelings and the changing my actions and managing my problems strategies for adolescents | Roleplays de-emphasized in the current adaptation for adolescents  Visualization activity replaced the roleplay for Adolescent Activity 4.3 (Changing my actions) | FGDs | Moderate |
| 24 | Treatment delivery: Surface adaptations: Content  or  Community needs, stigma, and context: Specific needs and other relevant contextual information: Group dynamics | Adolescent sessions typically start with icebreakers that involve adolescents singing songs or playing games deemed a bit too young for the 10-15 year old age group in NYC | Use of multimedia in session openings. Pop culture video clips and activities pertaining to the lessons learned in previous adolescent sessions and the upcoming session are recommended to illustrate concepts such as how big feelings can interfere with your goals |  |  |
| 25 | Treatment components: Framing treatment goals: Community-building  Or  Treatment delivery: Delivery format: Promotion of use | Adolescents wrap up their participation in EASE after completion of 7 sessions | Create optional post-EASE drop-in sessions integrated into existing community organization or afterschool programs, where adolescents can build community while practicing EASE skills. | FGDs | Moderate |

# Supplemental File 2 - Cognitive interviews

| **Box 1.** Cognitive interview questions.   - What are some things you would recommend to make adolescents more comfortable with sharing? How do we make sure that what you’re sharing is protected and respected? - Which activity was easiest for you to pay attention to? What part of the workshop made you feel most engrossed/engaged? - What were your expectations coming into these workshops and has your perspective changed since the beginning? - How have you grown since the beginning of the workshops? - What have you enjoyed about participating in this project? - Why do we need resources to support adolescents’ mental health? - How might EASE improve adolescents’ mental health? - How do you envision adolescent mental health to look like in the future? What are some strategies you think might improve adolescent mental health in New York City? |
| --- |

| **Box 2.** Quotes from cognitive interviews and FGDs with adolescents.  “EASE was helpful to learn how to control and understand your emotions, it’s not something a lot of people learn how to do because of society.”  “My favorite activity is the one with the stairs, it helps you step toward your goals.”  “We got stuck in some parts of the maze, there were a lot of dead ends, but you can still turn around and try another way to solve your problem.”  “My favorite was the body map, because it shows you how your emotions are related to your body, and how your emotions affect your body.”  “I knew what a vicious cycle was, but I never had a name for it.”  “When we were sitting down and just started deep breathing, it felt kind of weird, because I don’t regularly slow breathe.”  “The feelings pot was helpful in learning how not to have your feelings get to you. Don’t bottle up your emotions.” |
| --- |

### 
